# Verruciform Xanthoma of the Buccal Mucosa: A Case Report Highlighting a Rare Architectural Variant and Diagnostic Challenge

**DOI:** 10.1155/crid/1481564

**Published:** 2026-07-23

**Authors:** Bikash Shah, Toniya Raut, Pragya Regmee, Shashi Keshwar, Neetu Jain

**Affiliations:** ^1^ College of Dental Surgery, B.P. Koirala Institute of Health Sciences, Dharan, Nepal, bpkihs.edu; ^2^ Department of Oral Pathology, B.P. Koirala Institute of Health Sciences, Dharan, Nepal, bpkihs.edu; ^3^ Department of Oral Medicine and Radiology, B.P. Koirala Institute of Health Sciences, Dharan, Nepal, bpkihs.edu

**Keywords:** areca nut, case report, foam cells, oral verruciform xanthoma, verruciform xanthoma

## Abstract

Verruciform xanthoma (VX) is a relatively uncommon, benign, reactive lesion of the oral mucosa, representing only 0.025%–0.05% of all oral pathology cases. Clinically, it often presents as an asymptomatic, solitary, yellowish‐white, or red growth with a corrugated or verrucous surface. Because it frequently mimics verruco‐papillary lesions, clinical diagnosis is achieved in only approximately 2% of cases. Consequently, histopathological evaluation remains the “gold standard,” characterized by the pathognomonic accumulation of lipid‐laden foamy macrophages (xanthoma cells) within the connective tissue papillae. This report highlights a rare presentation of VX on the buccal mucosa, a site involved in only 8.5% of documented cases. Notable findings in this case included a 10‐year habit history of areca nut consumption. Rather than acting as a definitive etiological trigger, this habit served as a significant diagnostic confounder, elevating clinical suspicion of a malignant process. Histopathology confirmed the “flat” architectural variant, which is characterized by endophytic proliferation. Given the excellent prognosis following conservative surgical excision, this case emphasizes the need to include VX in the differential diagnosis of verrucous lesions to avoid overtreatment. The patients remained disease‐free, with no evidence of recurrence during follow‐up.

## 1. Introduction

Verruciform xanthoma (VX) is an uncommon, benign, and reactive lesion of the oral mucosa that was first documented by Shafer in 1971. In addition to oral involvement, its evidence is also documented on the genital mucosa (vulva and penis) and the skin surrounding the thigh and perineum. Oral VX remains a rare finding, representing only 0.025%–0.05% of all the pathologies. Although the etiopathogenesis of VX is not yet fully understood, it is thought to be a reactive change in response to squamous cell damage that triggers a macrophage response, giving it its histopathological characterization [1].

Oral VX predominantly affects middle‐aged and elderly individuals, with varying predilection for male and female as per the literature. It primarily affects the masticatory mucosa with gingiva as a most common site. The lesion typically presents as an asymptomatic, solitary, growth, or elevated plaque with verruciform texture which might be sessile or pedunculated. The size often varies from 2 mm to 1.5 cm whereas the color varies from yellow‐white to pink or red [1–3]. The clinical presentation often mimics several mucosal verruco‐papillary lesion‐like as squamous papilloma, verruca vulgaris, fibroma, Fordyce granules, leukoplakia, verrucous carcinoma, and squamous cell carcinoma [3, 4]. Hence, histopathological evaluation remains gold standard for diagnosis and ruling out other differentials. Here, we present a histopathologically proven case of VX in the buccal mucosa of a 55‐year‐old female, with a habit history of chewing areca nut. This case report is reported according to CARE checklist 2013 [5].

## 2. Case Presentation

**Figure 1:**
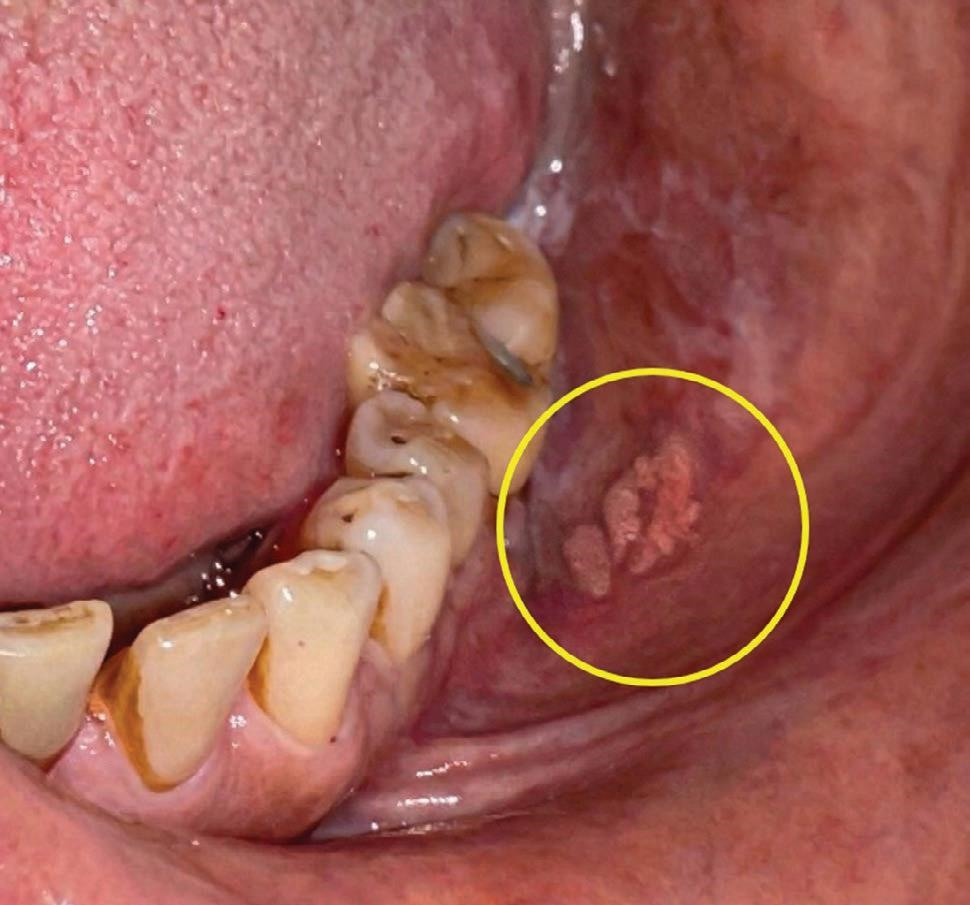
Clinical presentation: yellowish white growth on buccal mucosa (within yellow circle).

A 55‐year‐old female patient presented to the Department of Oral Medicine and Radiology for the evaluation of an asymptomatic plaque on left buccal mucosa. The patient had a habit of consuming areca nut for 10 years (3–4 times daily) and was under medication for hypertension for 1 year. On clinical examination, there was a single, soft, sessile, and oval‐shaped growth. The lesion was well‐demarcated with a verrucous surface. It was nontender and showed no signs of induration. It was present on the left buccal mucosa toward the vestibular depth in relation to 36, approximately measuring 2 × 2 cm in maximum dimension. Within the growth cluster of yellowish white nodules was observed. The adjacent surrounding mucosa showed brownish pigmentation (Figure 1), with a clinical diagnosis of verrucous hyperplasia and VX; an excisional biopsy was performed under local anesthesia. The obtained tissue specimen was fixed on 10% neutral buffer formalin and sent for routine histopathological examination. The macroscopic details were recorded to be a single soft tissue specimen, white to brown in color, firm in consistency, and measuring 22 × 22 × 5 mm in maximum dimension. Microscopic examination showed, parakeratotic, hyperplastic stratified squamous epithelium with endophytic growth, parakeratin plugging, and variably elongated rete ridges at a uniform depth. The entrapped connective tissue core within the hyperplastic epithelium in the lamina propria shows accumulation of numerous large, oval‐shaped xanthoma cells with central round basophilic nucleus, and granular, foamy cytoplasm. The underlying connective tissue showed moderate to dense distribution of lymphoplasmacytic infiltrates and areas of neutrophilic infiltration on the surface epithelium. (Figure 2) The histopathological presentations along with clinical features were diagnostic for oral VX. The patient remained disease‐free for 5 months after excision, with no local recurrence detected during follow‐up. Written informed consent was obtained from the patient.

Figure 2(a) Epithelium with variable elongation at uniform depth, 4x: (b) xanthoma cell within the lamina propria, entrapped core connective tissue, 10x inset. Xanthoma cell with granular and foamy cytoplasm, 40x H&E.

**Figure:**
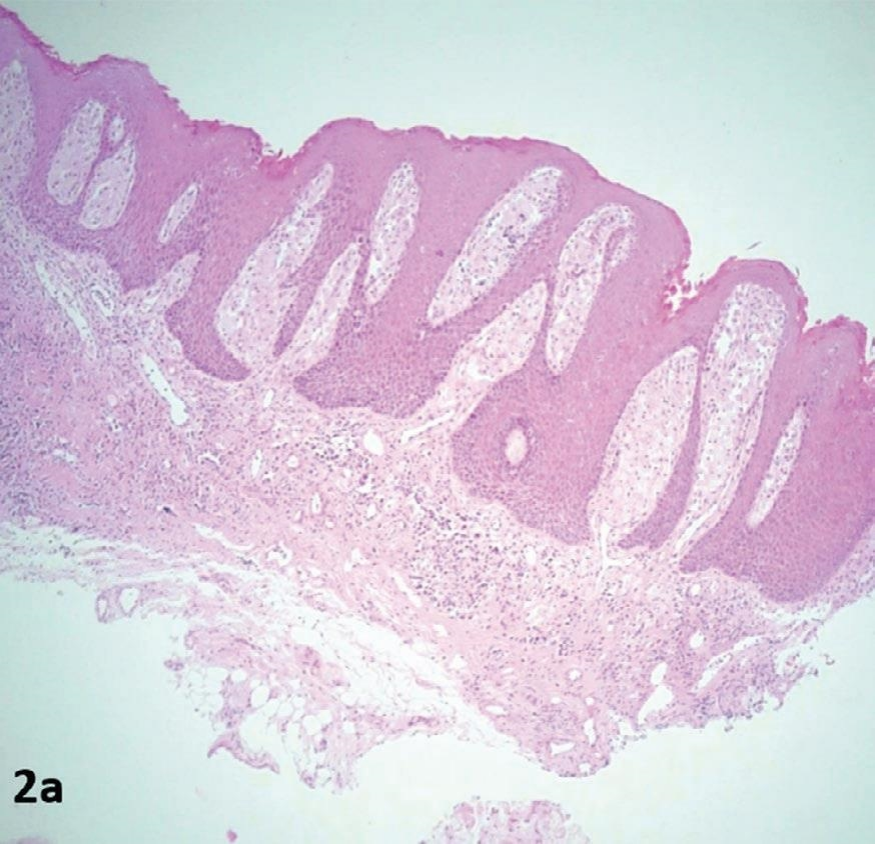
(a)

**Figure:**
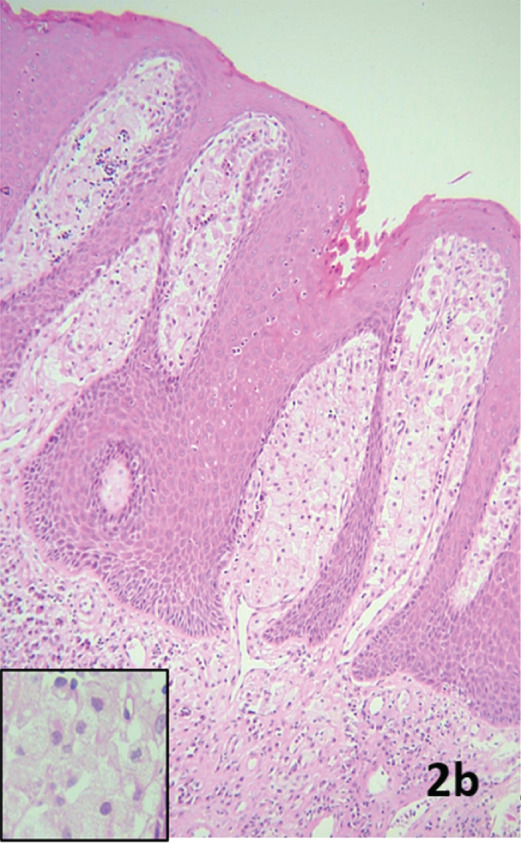
(b)

## 3. Discussion

VX is a relatively uncommon reactive lesion condition of oral cavity [1]. Although the gingiva remains the primary site of occurrence (51.2%), involvement of the buccal mucosa is considerably uncommon, representing 8.5% of documented cases whereas other sites includes the tongue, vestibule, lip, and floor of the mouth, with one case reported at an unspecified location [6]. The peak incidence of VX is seen in fifth and sixth decade of life which corresponds to the present case which was seen in 55 years of age. Gender demographics for this condition vary significantly across the literature. A large‐scale series by Belknap et al. [6] suggests a nearly balanced distribution with a female‐to‐male ratio of 1.06:1. Conversely, a recent review by Choudhari et al. [3] identified a much strong male predilection with male to female ratio of 3:1. Despite immunoprofile and molecular composition based advanced studies on VX, the etiopathogenesis still remains debated. As cited by Belknap et al. [6], Zegarelli proposed that foamy macrophage accumulation occurs when lipids released from degenerating epithelial cells are subsequently phagocytosed [6]. Conversely, as discussed by Belknap et al. [6], Nowparast proposed that the characteristic papillary and verrucous architecture of the epithelium are a secondary response to the presence of foam cells, which alter epithelial metabolism and induce hyperkeratotic changes. Both the hypotheses support a mutual interaction among macrophages, keratinocytes, and chronic inflammation. As noted by Hegde et al. [4], Zegarelli proposed that VX originates from an inflammatory reaction to local trauma or irritation, which facilitates the recruitment of lipid‐laden macrophages into the connective tissue. In the present case, the patient′s decade‐long history of areca nut consumption is a highly notable clinical factor. However, instead of being the primary etiological factor, this habit functioned mainly as a significant diagnostic confounder. As noted by Preto et al. [7], the presence of high‐risk behaviors such as tobacco or areca nut consumption frequently predisposes clinicians to suspect oral potentially malignant disorders or malignant lesions, potentially resulting in the misinterpretation or under recognition of benign reactive entities such as VX.

The present case offers valuable clinical insight into the morphological presentation of VX, which was definitively confirmed by the histopathological presence of xanthoma cells. In the absence of a pathognomonic clinical presentation, it may be challenging to include VX in the list of differentials. Hence, this report intends to emphasize the thorough evaluation of any verrucous appearing lesion. The report also emphasizes that presence of habit like areca nut or tobacco might encourage to think over its association with potentially malignant or malignant lesions, underestimating the possibilities of a reactive lesion like VX.

In 1981, Nowparast et al. classified oral VX into three architectural subtypes based on their low‐power appearance. Although these subtypes mimic other common oral lesions, the presence of xanthoma cells is a definitive diagnostic marker [6] (Table 1).

**Table 1:** Histological subtypes of verruciform xanthoma with corresponding morphological features and differential diagnoses.

Subtype	Histologic features	Common mimics
(a) Verrucous	Elevated and hyperkeratotic; features deep parakeratin crypts and a “cup‐shaped” appearance.	Verruca vulgaris, verrucous carcinoma
(b) Papillary	Finger‐like projections with connective tissue cores, extend above the mucosal surface.	Squamous papilloma, inflammatory papillary hyperplasia
(c) Flat	Proliferation occurs below the surface, featuring slender, uniform rete ridges, and minimal keratin.	Fibroma, lichen planus, nonverrucoid dysplasia

Our case represents the flat variant of VX, which is even uncommon among the three histopathological variants [8].

Establishing a clinical diagnosis of oral VX may be exceptionally challenging in the absence of a distinctive pathognomonic presentation. This difficulty was underscored by a major clinicopathological study of 212 cases by Belknap et al. [6], where a correct clinical suspicion was documented in only approximately 2% of the cases. Because it is yellow‐white, corrugated appearance often mimics conditions such as squamous papilloma, verruca vulgaris, fibroma, leukoplakia, verrucous carcinoma, and squamous cell carcinoma, histopathological evaluation remains the gold standard for diagnostic confirmation [3]. Once identified, VX is effectively managed through conservative surgical excision, which typically yields a superior prognosis [3]. Several alternative therapeutic approaches have been described, including CO_2_ laser ablation, cryotherapy, imiquimod application, topical corticosteroids, electrosurgery, and radiotherapy [9]. Recurrence following surgical excision appears to be infrequent, with only six cases (12%) reported in the literature and a mean recurrence interval of 2.8 ± 1.5 years [10].

## 4. Conclusion

VX presents a significant diagnostic challenge, particularly in the uncommon buccal mucosa. This case documents a rare flat architectural variant, emphasizing VX as an essential consideration in differential evaluation of verrucous lesions. Chronic areca nut consumption served as a diagnostic confounder, increasing clinical suspicion of malignancy and potentially diverting attention from the possibility of a benign reactive lesion. Given VX′s benign nature, histopathological identification of pathognomonic xanthoma cells remains the gold standard to prevent clinical overdiagnosis and unnecessary aggressive treatment of this lesion, which frequently mimics malignant conditions.

## Author Contributions

Bikash Shah: Conceptualization, Resources, Investigation, Writing Original Draft, and Writing Review and Editing. Toniya Raut: Conceptualization, Supervision, Resources, Writing Original Draft, and Writing Review and Editing. Pragya Regmee, Shashi Keshwar, and Neetu Jain: Writing Review and Editing.

## Funding

No funding was received for this manuscript.

## Ethics Statement

This is a single anonymized case report with informed consent. As it did not intervene with the patient′s treatment plans, institutional ethical approval was exempted.

## Conflicts of Interest

The authors declare no conflicts of interest.

## General Statement

Peer Review

Not commissioned, externally peer‐reviewed.

## Data Availability

The data that support the findings of this study are available from the corresponding author upon reasonable request.
